# A family of synthetic riboswitches adopts a kinetic trapping mechanism

**DOI:** 10.1093/nar/gku262

**Published:** 2014-04-29

**Authors:** Dennis M. Mishler, Justin P. Gallivan

**Affiliations:** Department of Chemistry, Emory University, 1515 Dickey Drive, Atlanta, GA 30322, USA

## Abstract

Riboswitches are sequences of RNA that control gene expression via RNA–ligand interactions, without the need for accessory proteins. Riboswitches consist of an aptamer that recognizes the ligand and an expression platform that couples ligand binding to a change in gene expression. Using *in vitro* selection, it is possible to screen large (∼10^13^ members) libraries of RNA sequences to discover new aptamers. However, limitations in bacterial transformation efficiency make screening such large libraries for riboswitch function in intact cells impractical. Here we show that synthetic riboswitches function in an *E. coli* S30 extract in a manner similar to how they function in intact *E. coli* cells. We discovered that, although this family of riboswitches regulates the initiation of protein translation, the fate of whether an RNA message is translated is determined during transcription. Thus, ligand binding does not bias a population of rapidly equilibrating RNA structures, but rather, co-transcriptional ligand binding kinetically traps the RNA in a conformation that supports efficient translation. In addition to providing new insights into the mechanisms of action of a family of synthetic riboswitches, our experiments suggest that it may be possible to perform selections for novel synthetic riboswitches in an *in vitro* system.

## INTRODUCTION

The ability to precisely control bacterial gene expression is critical for experiments in both basic and applied microbiology. Synthetic riboswitches are attractive tools for controlling gene expression because the level of expression is regulated in a ligand-dependent fashion (1,2). Riboswitches consist of an aptamer domain that recognizes the ligand, and an expression platform that modulates gene expression. Naturally occurring riboswitches are common in bacteria and regulate the synthesis of various metabolites (3,4). The ligands recognized by natural aptamer domains include amino acids, nucleotide derivatives, coenzymes, and even ions (3). *In vitro* selection techniques make it possible to screen large libraries of RNA (>10^13^ members) to discover aptamers that bind to novel ligands both tightly and selectively (5,6). We and others have shown that it is possible to convert known aptamers, such as the theophylline aptamer, into functional riboswitches through high-throughput *in vivo* screening or rational design (7–13). However, both *in vivo* screening and rational design have limitations.

Rational design of riboswitches is often based on methods for computing the relative thermodynamic stabilities of competing RNA structures (14–17). While a variety of computational methods, such as *mfold* (18) and Vienna RNA (19), can reasonably predict the relative ground-state free energies of different RNA conformations, they are less able to predict the structures and free energies of the intermediates and transition states that separate the conformations. If the kinetic barriers between conformations are sufficiently low, thermodynamic (equilibrium) models for riboswitch function should work well. However, if there are significant kinetic barriers between riboswitch conformations, equilibrium models that fail to account for these barriers will likely fail. To determine if these computational predictions are accurate, the predicted riboswitches must be tested in a biologically relevant system, a few sequences at a time.

Synthetic riboswitches have also been discovered using high-throughput screens or selections in cells (11,20–22). Such methods are attractive because they neither require prior structural knowledge of the aptamer, nor presuppose a mechanism of action for the resulting riboswitch(es). While the best genetic selections performed in *Escherichia coli* can assay library sizes on the order of 10^8^ members (21,23), a limitation imposed by the transformation efficiency, such libraries are considerably smaller than those that may be assayed in a cell-free system. By comparison, most aptamers are discovered using *in vitro* selections, which are capable of assaying libraries on the order of 10^13^ members (5,6).

Because cell-free systems can sample much larger library sizes than cell-based systems (>10^13^ versus 10^8^ members), it is tempting to think that it may be possible to directly select for synthetic riboswitches *in vitro* (24), much in the way that aptamer selections are performed today. However, since many applications of riboswitches will ultimately require intact cells, it is also important to establish that riboswitches function similarly both *in vitro* and in cells (25).

**Figure 1.**
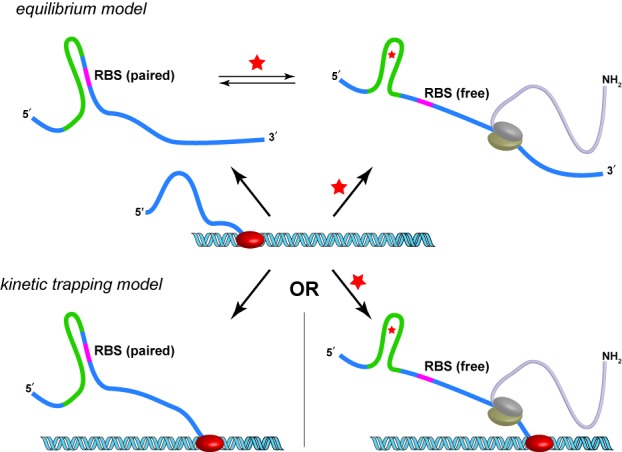
Models for riboswitch function. In the equilibrium model (top), a newly transcribed RNA can adopt a conformation in which the ribosome binding site (RBS) is paired and translation is inefficient. This conformation is in equilibrium with a conformation in which the RBS is free (efficient translation). The presence of theophylline (red star) shifts the equilibrium to favor the RBS-free conformation. In the kinetic trapping model (bottom), in the absence of theophylline, the nascent RNA adopts a conformation in which the RBS is paired and translation is inefficient. If theophylline is present during transcription, the theophylline-bound RNA adopts a conformation in which the RBS is free and translation is efficient. In the kinetic trapping model, the free and bound structures do not equilibrate on the timescale of the experiment.

Here we show that riboswitches expressed in *E. coli* S30 extract function in a manner similar to the way they function in *E. coli* cells. To our surprise, it was critical that transcription was active for the vast majority of riboswitches to function well in extract. Our results indicate that, at least in extract, many of these riboswitches do not act as rapidly equilibrating systems, but rather as kinetic traps that require the presence of ligand during transcription (Figure 1). These findings highlight the utility of cell-free extracts in the design and discovery of new synthetic riboswitches and the characterization of known riboswitches.

## MATERIALS AND METHODS

### General considerations

All plasmid manipulations utilized standard cloning techniques and all constructs were verified by DNA sequencing. Purification of plasmid DNA, PCR products and enzyme digestions were performed using kits from Qiagen. Oligonucleotides were purchased from IDT.

### Cloning

The luciferase constructs derived from the switch pack sequences (26) were created using standard cloning procedures. The specific riboswitch sequences can be found in Supplementary Figures S5 and S6. The firefly luciferase gene was derived from the plasmid pBESTluc (Promega) using forward primer DMM141 (see below for sequence) and reverse primer DMM157. Overlap PCR was then performed using this PCR product and PCR products derived from the 5′ UTR sequences in Supplementary Figure S5 as templates. The primers for the overlap reactions were forward primer DMM161, which contains the tac promoter sequence, and reverse primer DMM157.

PCR products were purified using a PCR purification kit (Qiagen) and then digested with BamH I and Pst I, underlined below, and gel purified using a gel extraction kit (Qiagen). Digested product was ligated into a pUC19 vector, followed by butanol precipitation and resuspension with 20 μl water. TOP10 cells were transformed with the ligation products via electroporation, plated on LB agar with ampicillin, and grown overnight at 37°C. Colonies were picked the next day and grown in LB liquid media with ampicillin overnight at 37°C. Plasmid DNA was isolated from these overnight cultures via miniprep kit (Qiagen). DNA sequencing confirmed the identities of these plasmids. All cloning enzymes used above were obtained from NEB. Luciferase reporters for the D family riboswitches (Supplementary Figure S6) were created using the same procedures and primers.

DNA templates for *in vitro* transcription were generated from the above plasmids using forward primer DMM107, which contains the T7 promoter sequence, and reverse primer DMM157.

Primers mentioned above are as follows:

DMM107: 5′-TAATACGACTCACTATAGGGACTCACTATAGGTACC-3′
DMM141: 5′-ATGGAAGACGCCAAAAACATAAAGAAAGGCCCGG-3′
DMM157: 5′-ACGATCTGCAGCCGCACACCAGTAAGGTGTGCGGTTACAATTTGG-3′
DMM161: 5′-AGTCACGGATCCGAGCTGTTGACAATTAATCATCGGCTCGTATAATGTGTGGG-3′.

### Luciferase assays

*In vivo luciferase reactions*: saturated overnight cultures of *E. coli* TOP10 cells containing a pUC19 plasmid with a luciferase reporter under the control of a riboswitch were diluted 1:500 in fresh LB/AMP media and allowed to grow to mid-exponential phase. Culture was then prepared for luciferase measurements by placing the reactions on dry ice and lysing with 1x CCLR (Promega) following the manufacturer's procedure.

Run-off *in vitro* transcription reactions were performed using the AmpliScribe T7-Flash kit (Epicentre) per the manufacturer's procedure with ∼1 μg of DNA template. After 30 min at 37°C, reactions were treated with DNase I for 15 min and then precipitated with 5 M NH_4_OAc, as described in the AmpliScribe protocol.

*In vitro* transcription and/or translation reactions were performed using S30 *E. coli* extract (Promega, item numbers L1020 and L1030). Reaction conditions followed those recommended by the manufacturer, and included S30 premix, S30 extract, amino acids, template DNA or RNA, and either theophylline or water. An amount of 40 ng/μl of plasmid DNA template or 800 ng/μl of *in vitro* transcribed RNA template was used for each reaction. Theophylline concentrations were typically 2 mM, except for the dose response curves. Reaction volumes varied from 10 to 50 μl, but results were reproducible regardless of total volume. Reactions were incubated at 37°C for 30 min, unless otherwise noted. Reactions were quenched by placing them on ice with dilution buffer provided with the S30 extract per the manufacturer's instructions.

Luciferase assays were conducted using samples from *in vitro* translation or cell cultures. In each case, ∼20–30 μl of sample was then added to a well on a black opaque 96-well plate. An equivalent amount of luciferase assay reagent (Promega) was added to each well and mixed. Luciferase activity was detected by reading the 96-well plates with a Biotek Synergy HT plate reader, using the luminescence procedure with a sensitivity of 150 for most experiments.

*Presentation of the luminescence data:* *In vivo* luciferase expression data were initially normalized relative to OD_600_ values at the time of harvesting. For *in vitro* translation reactions, the data were normalized relative to the amount of DNA or RNA template used. These values were then normalized relative to a single control reaction or time point within that experiment. More specifically, the values of each reaction or time point were divided by the value of the single control reaction or time point, generating a normalized scale of 0–100%. Normalized expression data were then averaged between at least three independent experiments. Standard deviations were determined and graphed as error bars. From these average expression values, estimated activation ratios (ARs) were determined and presented. Specifically, an AR is calculated by dividing the amount of gene expression in the presence of theophylline by the amount of gene expression in the absence of theophylline.

Dose response curves were conducted *in vivo* or in S30 extract as described above, but with varying amounts of theophylline as shown in the figures.

### Decoupled transcription/translation reactions in S30 extract

*In vitro* transcription/translation reactions in S30 extract with rifampicin and 40 ng/μl of DNA template were conducted as described above with the following exceptions. Amino acids were omitted during the initial 5 min in all cases. After 5 min (the transcription-only phase), a solution of rifampicin in water (2.5 mg/ml) was added, yielding a final reaction concentration of 250 μg/ml rifampicin. One minute later, amino acids were added to the reaction as well as additional water and/or theophylline to reach a final concentration of either no theophylline or 2 mM theophylline. The reaction (now in the translation-only phase) proceeded for the amount of time indicated followed by quenching as described above for *in vitro* luciferase assays.

### Secondary structure models

Supplementary Figures S5 and S6 and the estimated ΔG for riboswitch sequences were generated using *mfold* (18), specifically the Quikfold option for RNA, using the entire 5′ UTR and the first two codons of the luciferase reporter. Inclusion of additional codons did not appear to disrupt the structures shown here.

## RESULTS

**Figure 2.**
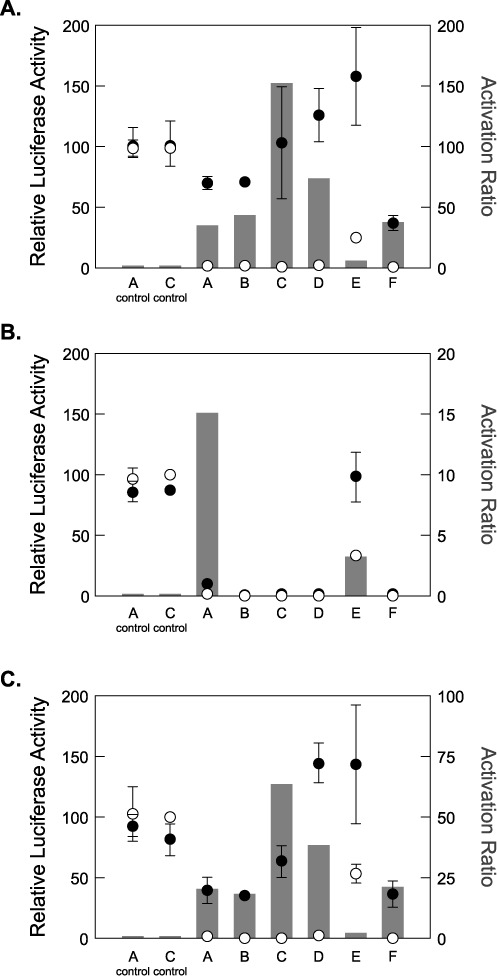
(**A**) Performance of riboswitches expressed in intact *E. coli* cells. Left axis: luciferase activity in the absence (open circles) or presence (filled circles) of 2 mM theophylline. Values are normalized to the C control in the absence of theophylline. The ‘A control’ and ‘C control’ have identical ribosome binding site sequences to riboswitches A and C, respectively, but lack the theophylline-binding aptamer. Error bars reflect the standard deviation for three independent determinations. Right axis: the activation ratio, which is expressed as the relative luciferase activity in the presence of 2 mM theophylline divided by the activity in the absence of theophylline. (**B**) Performance of riboswitches expressed in *E. coli* S30 extract (RNA templates). Note that the right y-axis range has been changed by an order of magnitude to better reveal the smaller activation ratios. (**C**) Performance of riboswitches expressed in *E. coli* S30 extract (DNA templates). Note that the right y-axis range has been changed, relative to panel (A), by a factor of 2. Data for (B) and (C) are presented as in (A).

We first tested whether a set of previously reported theophylline-responsive synthetic riboswitches could control the expression of a luciferase reporter gene in intact *E. coli* cells (26,27). These riboswitches were selected because they were previously published and are frequently requested by other laboratories (26,27). We created six plasmids (A–F) in which the *tac* promoter was used to drive the expression of a luciferase reporter gene under riboswitch control. *E. coli* cells were transformed with one of the plasmids, grown on selective solid media, and then in selective liquid media either in the presence or absence of theophylline. As shown in Figure 2A, cells harboring the riboswitch-controlled luciferase reporter exhibit theophylline-dependent increases in luciferase activity in the presence of 2 mM theophylline. Cells containing the luciferase reporter alone show minimal theophylline-dependent changes in luciferase activity. The behavior of these riboswitches, including their ARs, with the luciferase reporter gene mimics that previously observed with the β-galactosidase reporter gene (26), although the E switch does exhibit differences that may result from sequence-specific interactions between the riboswitch and one or both of the reporter genes. These data indicate that our series of riboswitch-controlled luciferase reporters regulate gene expression in a theophylline-dependent manner *in vivo*.

Previous results from our lab suggested that related riboswitches functioned via a post-transcriptional mechanism, in which theophylline binding biases equilibrating RNA structures toward conformations that favor translation (Figure 1) (22). However, we were unable to fully rule out competing mechanisms of riboswitch function. Given these findings, we anticipated that mRNA templates produced by *in vitro* transcription would yield theophylline-dependent changes in luciferase activity when added to an *in vitro* translation system.

To test this, we placed the DNA constructs described above under control of a T7 promoter to drive the expression of the riboswitch-luciferase reporter gene. mRNA was produced by *in vitro* transcription, and the purified transcripts were used as templates for *in vitro* translation reactions in *E. coli* S30 extract. Translation reactions were performed either in the absence or presence of 2 mM theophylline (Figure 2B). Surprisingly, all of the riboswitches showed little-to-no theophylline-dependent increases in luciferase activity. Only the E switch with the luciferase reporter gene showed an AR (∼3-fold) similar to its *in vivo* AR (∼6-fold). In contrast, a control mRNA lacking a riboswitch showed robust luciferase activity both in the presence and absence of theophylline, demonstrating that the S30 system is capable of efficiently translating mRNA produced by *in vitro* transcription (Figure 2B). The quality of the mRNA templates was confirmed via agarose gel (Supplementary Figure S1). mRNA templates were also heated to 65°C in the presence of the ligand and then cooled prior to *in vitro* translation in extract. Surprisingly, we observed no ligand dependent changes in luciferase activity (Supplementary Figure S2).

Since *in vitro* transcribed riboswitch mRNA templates functioned poorly in S30 extract, we asked whether DNA templates actively transcribed to mRNA in S30 extract would function as riboswitches. To test this, we introduced the same plasmid DNA previously used in *E. coli* cells into S30 extract that is competent for both transcription and translation. As depicted in Figure 2C, DNA templates produce theophylline-dependent increases in luciferase activity in S30 extract. This is in stark contrast to the results seen when mRNA templates were used. Although the ARs observed in extract are approximately 2-fold lower than those seen in cells, the riboswitches with the highest ARs retain this distinction both *in vivo* and in extract (compare Figure 2A–C).

**Figure 3.**
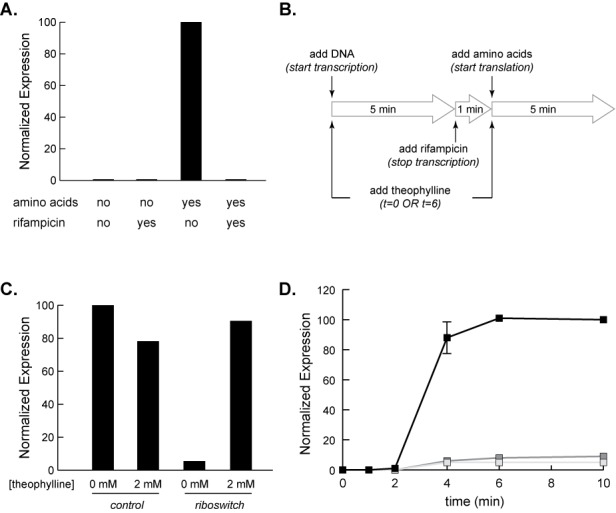
Decoupling transcription and translation in *E. coli* S30 extract. (**A**) Luciferase activity of the D riboswitch added to the S30 extract as a DNA template. Expression is only observed when rifampicin is absent (transcription is active) and amino acids are present (translation is active). (**B**) Schematic of decoupling transcription and translation in S30 extract. Theophylline (2 mM) is added at *t* = 0 min (present for transcription and translation) or *t* = 6 min (present for translation only). (**C**) Luciferase activity for a DNA template of the D riboswitch (or aptamer-less control) added to S30 extract using the decoupling procedure shown in (B). 2 mM theophylline is added at *t* = 0 min (present for transcription and translation). Data are normalized to the relative luminescence of the control with 0 mM theophylline. A single data set is presented. (**D**) Decoupled transcription and translation of the luciferase reporter under control of D switch performed as described in panel (B). Aliquots were removed at the times indicated after addition of amino acids. Three separate time courses were performed: no theophylline added (light gray squares), 2 mM theophylline added only after transcription was terminated (dark gray squares), and 2 mM theophylline present during transcription and translation (black squares). Data are normalized to the relative luminescence of the highest 10 min data point. Each data point is the average of three independent experiments. Error bars show standard deviation.

Our data suggest that active transcription is important for riboswitch function in S30 extract, while our previous studies suggested that active transcription is not essential for riboswitch function in cells (22). To further explore the relative contributions of transcription and translation in extract, we decoupled the two processes. This was accomplished by adding a DNA template to the transcription/translation extract either in the absence of amino acids (Figure 3A, first bar), or in the presence of a transcription inhibitor, rifampicin (Figure 3A, last bar). Only when both transcription and translation can occur do we see luciferase activity. If either transcription or translation is inhibited, through omission of amino acids or by addition of rifampicin, no luciferase activity is observed. In Figure 3B, we present a schematic of the decoupling procedure we use: after 5 min of reaction time in the absence of amino acids, during which RNA is transcribed but not translated, we added the transcription inhibitor rifampicin (22). After waiting for one additional minute, we added a mixture of the 20 canonical amino acids to allow translation, while transcription initiation is blocked. Control luciferase activity generated using this procedure is presented in Figure 3C. Theophylline was added either at the start of transcription, at the start of translation, or not at all, allowing us to determine the importance of ligand either during or after transcription. The results of the time course experiments are shown in Figure 3D.

As expected, the reaction lacking theophylline shows very little increase in luciferase activity over time. To our surprise, the reaction that had theophylline present only during translation also shows little increase in luciferase activity. This stands in contrast to the reaction in which theophylline was present during both transcription and translation. In this case, we observe robust theophylline-dependent increases in luciferase activity. Even after accounting for the extra time that theophylline is present in the transcription/translation reaction (11 min) versus translation only (5 min), there is a significant increase in signal that results from having theophylline present while the mRNA is being transcribed. To confirm that this difference was not simply a result of the time difference described above, we extended the translation period for a total of 30 min and found identical behavior (Supplementary Figure S3). These results suggest that to achieve the highest ligand-dependent changes in gene expression in this *in vitro* system, it is critical to have ligand present during transcription (Figure 3D and Supplementary Figure S4). However, our previous mechanistic studies carried out in intact cells (22) suggested that there is also a post-transcriptional component to riboswitch function.

To reconcile these observations, we hypothesized that these synthetic riboswitches can function through two competing mechanisms: (i) a co-transcriptional kinetic-trapping mechanism, in which it is critical to have ligand present while transcription is active, and (ii) an equilibrium mechanism in which mRNA structures equilibrate rapidly. We speculated that riboswitches with higher background levels of protein expression would have a greater contribution from the equilibrium mechanism. To test this, we studied a series of closely related riboswitches derived from the D switch. These sequences are identical with the exception of an 8 nt sequence between the aptamer and the start codon (Table 1).

**Table 1.** D family of riboswitches: names, variable sequence, free energy of the 5′ UTR (kcal/mol), free energy of the sequester helix (SH, kcal/mol), and observed activation ratios either *in vivo* or as an RNA template in extract (Supplementary Figure S7)

Construct	Sequence	−ΔG 5′ UTR	−ΔG SH	*In vivo* AR	*In vitro*_RNA_ AR
615	CACUUCUA	20	17	5	2
611	GGGCGCAG	20.9	17.9	7	3
612B	UGGGACAA	21.4	18.4	8	3
602A	AAAGGAUG	24.6	21.6	10	17
D switch	UAAGGUAA	25.5	22.5	90	3
607	UAUGAUGG	25.6	22.6	41	7
602B	GAAGAUGG	26.7	23.7	244	3
609	CGGGCGGG	30.1	27.1	141	2

**Figure 4.**
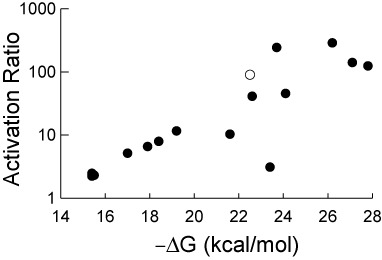
Plot of the observed activation ratios versus the *mfold*-predicted ΔG of the OFF state sequester helices for a set of ‘D family’ riboswitches. Each data point represents a single riboswitch sequence, including the parent D switch (open circle). The activation ratios were estimated using expression data that can be found in Supplementary Table S1.

The predicted secondary structures and free energies of the OFF states (Supplementary Figure S6) were generated using *mfold* (18). We tested these switches in growing *E. coli* cells, and then plotted the observed AR against the estimated −ΔG of the OFF state sequester helix (SH) for each riboswitch (Figure 4). The resulting plot shows a trend: as the predicted stability of the OFF state SH increases, the observed *in vivo* AR also increases. This result aligned with our expectations as riboswitches with higher ARs typically have lower gene expression in the absence of theophylline (22,28). Consistent with the observations of Figure 2B, the riboswitches that display the highest ARs when a DNA template is used, those that we would predict to have a dominant kinetic component, show minimal theophylline-dependent increases in gene expression when an mRNA template is used in extract (Table 1, Supplementary Figure S7). However, the riboswitches with lower ARs (and less stable OFF states), which we would predict to have a larger thermodynamic component, have comparatively higher levels of theophylline-dependent increases in gene expression when an mRNA template is used (Supplementary Figure S7), consistent with having a larger thermodynamic component.

**Figure 5.**
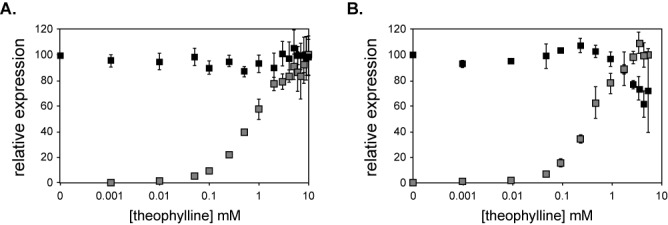
Dose response curves for luciferase reporters. (**A**) Dose–response curves for luciferase reporters expressed in intact *E. coli* cells. The gray squares correspond to the C riboswitch; the black squares correspond to a control lacking the aptamer. Data are normalized to the expression of either the C riboswitch in 10 mM theophylline or the control in 0 mM theophylline. Each data point represents the average of three independent measurements; error ± SD. (**B**) Dose–response curves for luciferase reporters expressed from DNA templates expressed in *E. coli* S30 extract. The gray squares correspond to the C riboswitch; the black squares correspond to a control lacking the aptamer. Data are normalized to the expression of either the C riboswitch in 6 mM theophylline or the control in 0 mM theophylline. The dose–response curve was terminated at 6 mM theophylline in extract because of the increasing toxicity of the ligand, as seen with the control dose response (black squares). Each data point represents the average of three independent measurements; error ± SD.

Finally, we used the opportunity of working in an *in vitro* system to address a long observed discrepancy between the reported *K*_d_ of ∼400 nM of the theophylline aptamer for its ligand (29,30) and the concentration of theophylline required to activate the riboswitch in intact *E. coli* cells (∼300–500 μM) (11,31). We performed dose–response relationships using the C-switch and a no-aptamer luciferase control both in intact *E. coli* cells and in extract. Surprisingly, the two dose–response curves obtained from extract and from cells are highly similar (Figure 5). The only significant difference occurs when the concentration of theophylline increases above 2 mM. In cells, luciferase activity begins to level off at 2 mM theophylline. However, in extract, luciferase activity increases until at least 6 mM theophylline, a concentration that greatly reduces the extract's ability to produce functional luciferase, as seen in the dose–response relationship for the no-aptamer control (Figure 5B). We and others (11,12) had previously interpreted the large difference between the *K*_d_ of the aptamer for theophylline and the concentration of theophylline required to elicit changes in gene expression in cells resulted from a lack of permeability of the cells to theophylline (or an active efflux mechanism). In our *in vitro* experiments, theophylline permeability is not an issue, yet we still observe that millimolar concentrations of theophylline are required to activate the riboswitches. This result is consistent with a kinetic trapping mechanism for riboswitch function, where the concentration of ligand at the time of initial folding determines the structure formed, not the amount of ligand present throughout the lifetime of the molecule.

## DISCUSSION

We began this study to determine whether theophylline-dependent synthetic riboswitches function *in vitro* in the same manner that they function in intact cells. We discovered that the function of riboswitches encoded as DNA templates and expressed in *E. coli* S30 extract closely mimics the function of these same riboswitches when expressed in intact *E. coli* cells. However, when these riboswitches were produced as mRNA templates by run-off transcription and then added to *E. coli* extract, they failed to show strong theophylline-dependent increases in gene expression. Our study of riboswitch function in extract demonstrates that the system serves as a good model for riboswitch function *in vivo*, can be used to gain a greater mechanistic understanding of synthetic riboswitches, and can likely be adapted to engineer novel riboswitches in a functionally relevant environment. Our results also suggest that *in vitro*, these riboswitches operate by a kinetic mechanism, in which the ‘decision’ to translate an mRNA is made co-transcriptionally.

These results were surprising to us because previous studies from our lab (22) suggested that riboswitches from this family were under thermodynamic (equilibrium) control. In those experiments, we showed that a reporter gene under the control of F switch in intact cells produced theophylline-dependent changes in gene expression in the absence of active transcription, although these changes were relatively small. It is important to note that the functional properties of F switch are not affected by the reporter gene sequence (Figure 2). The data shown in Figures 2 and 3 show the existence of an important kinetic component to riboswitch function, suggesting that our earlier studies did not tell the entire story.

Several of our previous observations of riboswitch function make better sense when viewed in light of these new data. For example, we previously observed that nuclease digestion experiments of theophylline-dependent riboswitches showed only modest ligand dependent changes in RNA structure (28). We had suggested that the modest changes resulted from a small fraction of functional RNA sequences appearing against the background of a larger population of non-functional RNAs. We now argue that kinetic factors, which would not be detectable in experiments lacking active transcription (e.g. SHAPE; T1 nuclease mapping), contribute greatly to the function of these riboswitches and that this explains why only modest changes were previously observed. The current data suggest that the equilibrium component is only a small contributor to the overall function of these riboswitches.

The present data also help explain why theophylline-dependent riboswitches expressed in cells typically respond to millimolar concentrations of ligand, while the aptamer binds theophylline at submicromolar concentrations *in vitro* (29,30). We previously attributed this difference to observations regarding theophylline metabolism and permeability in *E. coli* (32) that suggested that the intracellular concentration of theophylline relative to the medium may be ∼1000-fold lower (11). After correcting for this difference, it appeared that our earliest reported riboswitches acted under equilibrium conditions (11,22,31). However, several of our riboswitches display ARs that are inconsistent with a mechanism in which equilibrium phenomena dominate (26,28). In our *in vitro* experiments, the riboswitches show half maximal activation at nearly millimolar concentrations of theophylline. As ligand permeability is no longer an issue, these data further implicate a kinetic component to the mechanism.

Our data suggest that this family of theophylline-dependent synthetic riboswitches operates via a mechanism that includes both a kinetic component (which requires active transcription) and an equilibrium component (which doesn't require active transcription). However, the overall behavior of the riboswitches is dominated by the kinetic component. Our results are consistent with a recent study by Forster *et al.* that explored the importance of the kinetics of ligand binding in engineered tetracycline riboswitches (33).

Most synthetic riboswitches have been created from aptamers that were selected for their ability to bind a ligand tightly. It has also been speculated that natural riboswitches may be derived from aptamers that were selected for their ability to bind ligands tightly in an RNA world, but were later repurposed to function in riboswitches. Consistent with this suggestion, the aptamers from the flavin mononucleotide (FMN) (34) and cyclic-di-GMP riboswitches bind their ligands with equilibrium dissociation constants that are orders of magnitude lower than the ligand concentrations that the riboswitches sense in cells. The cyclic-di-GMP aptamer, for example, binds its ligand with a *K*_d_ of ∼10 pM. It achieves such tight binding because its dissociation rate is extremely slow (*t*_1/2_ ∼ 43 days) (35). As *E. coli* grown in rich medium divide approximately every 20 min and the average mRNA half-life is ∼6 min (36), this riboswitch cannot reach equilibrium, and thus acts as a kinetic trap like the theophylline riboswitches described here.

These observations suggest that when there is a strong kinetic component to riboswitch activity, the equilibrium dissociation constant of the aptamer may not accurately reflect riboswitch performance. In such cases, the ligand association kinetics may be a better predictor of riboswitch function. This has implications for aptamer selection and riboswitch design. For example, an aptamer may display tight binding that is the result of a very slow ligand dissociation rate. However, such an aptamer may perform very poorly in the context of a kinetically controlled riboswitch. We suggest that aptamers that maximize ligand association kinetics are better suited for kinetically controlled riboswitches.

The synthetic riboswitches reported thus far respond to only a small number of ligands. One of the biggest hurdles in the creation of synthetic riboswitches has been the discovery of new aptamers through *in vitro* selection that are appropriate for riboswitch function (2). Another hurdle is that aptamer selections are but one step in the process of discovering new riboswitches. Historically, aptamer selections have been carried out in cell-free systems that allow one to assay ∼10^13^ sequences, while screens and selections for riboswitch function have been carried out in cells, which limits library sizes to ∼10^8^ members. Our results and others (24,25) suggest that an *in vitro* system utilizing *E. coli* S30 extract recapitulates riboswitch function in intact cells. This opens the door to directly selecting new riboswitches from large (∼10^13^ members) libraries of RNA without performing a separate aptamer selection. We expect that it will soon be possible to directly select riboswitches using *in vitro* systems with the expectation that these riboswitches will readily function in intact cells.

## SUPPLEMENTARY DATA

Supplementary Data are available at NAR Online.

SUPPLEMENTARY DATA

## References

[1] Topp S., Gallivan J.P. (2010). Emerging applications of riboswitches in chemical biology. ACS Chem. Biol..

[2] Wittmann A., Suess B. (2012). Engineered riboswitches: expanding researchers’ toolbox with synthetic RNA regulators. FEBS Lett..

[3] Breaker R.R. (2011). Prospects for riboswitch discovery and analysis. Mol. Cell.

[4] Serganov A., Nudler E. (2013). A decade of riboswitches. Cell.

[5] Ellington A.D., Szostak J.W. (1990). In vitro selection of RNA molecules that bind specific ligands. Nature.

[6] Tuerk C., Gold L. (1990). Systematic evolution of ligands by exponential enrichment: RNA ligands to bacteriophage T4 DNA polymerase. Science.

[7] Werstuck G., Green M.R. (1998). Controlling gene expression in living cells through small molecule-RNA interactions. Science.

[8] Harvey I., Garneau P., Pelletier J. (2002). Inhibition of translation by RNA-small molecule interactions. RNA.

[9] Suess B., Hanson S., Berens C., Fink B., Schroeder R., Hillen W. (2003). Conditional gene expression by controlling translation with tetracycline-binding aptamers. Nucleic Acids Res..

[10] Buskirk A.R., Landrigan A., Liu D.R. (2004). Engineering a ligand-dependent RNA transcriptional activator. Chem. Biol..

[11] Desai S.K., Gallivan J.P. (2004). Genetic screens and selections for small molecules based on a synthetic riboswitch that activates protein translation. J. Am. Chem. Soc..

[12] Bayer T.S., Smolke C.D. (2005). Programmable ligand-controlled riboregulators of eukaryotic gene expression. Nat. Biotechnol..

[13] Malo N., Hanley J.A., Cerquozzi S., Pelletier J., Nadon R. (2006). Statistical practice in high-throughput screening data analysis. Nat. Biotech..

[14] Beisel C.L., Smolke C.D. (2009). Design principles for riboswitch function. PLoS Comput. Biol..

[15] Chen X., Ellington A.D. (2009). Design principles for ligand-sensing, conformation-switching ribozymes. PLoS Comput. Biol..

[16] Carothers J.M., Goler J.A., Juminaga D., Keasling J.D. (2011). Model-driven engineering of RNA devices to quantitatively program gene expression. Science.

[17] Quarta G., Sin K., Schlick T. (2012). Dynamic energy landscapes of riboswitches help interpret conformational rearrangements and function. PLoS Comput. Biol..

[18] Zuker M. (2003). Mfold web server for nucleic acid folding and hybridization prediction. Nucleic Acids Res..

[19] Gruber A.R., Lorenz R., Bernhart S.H., Neubock R., Hofacker I.L. (2008). The Vienna RNA websuite. Nucleic Acids Res..

[20] Topp S., Gallivan J.P. (2008). Random walks to synthetic riboswitches—a high-throughput selection based on cell motility. Chembiochem.

[21] Fowler C.C., Brown E.D., Li Y. (2008). A FACS-based approach to engineering artificial riboswitches. Chembiochem.

[22] Lynch S.A., Desai S.K., Sajja H.K., Gallivan J.P. (2007). A high-throughput screen for synthetic riboswitches reveals mechanistic insights into their function. Chem. Biol..

[23] Lynch S.A., Topp S., Gallivan J.P. (2009). High-throughput screens to discover synthetic riboswitches. Methods Mol. Biol..

[24] Martini L., Mansy S.S. (2011). Cell-like systems with riboswitch controlled gene expression. Chem. Commun. (Camb.).

[25] Lemay J.F., Desnoyer G., Blouin S., Heppell B., Bastet L., St-Pierre P., Masse E., Lafontaine D.A. (2011). Comparative study between transcriptionally- and translationally-acting adenine riboswitches reveals key differences in riboswitch regulatory mechanisms. PLoS Genet..

[26] Topp S. (2010). Synthetic riboswitches that induce gene expression in diverse bacterial species. Appl. Environ. Microbiol..

[27] Reynoso C.M., Miller M.A., Bina J.E., Gallivan J.P., Weiss D.S. (2012). Riboswitches for intracellular study of genes involved in Francisella pathogenesis. MBio.

[28] Lynch S.A., Gallivan J.P. (2009). A flow cytometry-based screen for synthetic riboswitches. Nucleic Acids Res..

[29] Jenison R.D., Gill S.C., Pardi A., Polisky B. (1994). High-resolution molecular discrimination by RNA. Science.

[30] Zimmermann G.R., Jenison R.D., Wick C.L., Simorre J.P., Pardi A. (1997). Interlocking structural motifs mediate molecular discrimination by a theophylline-binding RNA. Nat. Struct. Biol..

[31] Topp S., Gallivan J.P. (2007). Guiding bacteria with small molecules and RNA. J. Am. Chem. Soc..

[32] Koch A.L. (1956). The metabolism of methylpurines by Escherichia coli. I. Tracer studies. J. Biol. Chem..

[33] Forster U., Weigand J.E., Trojanowski P., Suess B., Wachtveitl J. (2012). Conformational dynamics of the tetracycline-binding aptamer. Nucleic Acids Res..

[34] Wickiser J.K., Winkler W.C., Breaker R.R., Crothers D.M. (2005). The speed of RNA transcription and metabolite binding kinetics operate an FMN riboswitch. Mol. Cell.

[35] Smith K.D., Lipchock S.V., Ames T.D., Wang J., Breaker R.R., Strobel S.A. (2009). Structural basis of ligand binding by a c-di-GMP riboswitch. Nat. Struct. Mol. Biol..

[36] Selinger D.W., Saxena R.M., Cheung K.J., Church G.M., Rosenow C. (2003). Global RNA half-life analysis in Escherichia coli reveals positional patterns of transcript degradation. Genome Res..

