# Supplementary material for: A family of synthetic riboswitches adopts a kinetic trapping mechanism
Source: Nucleic Acids Res. 2014 Apr 29;42(10):6753–61. doi: 10.1093/nar/gku262 (PMC4041436; doi:10.1093/nar/gku262)
Supplement: SUPPLEMENTARY DATA [file supp_42_10_6753__index.html]

A family of synthetic riboswitches adopts a kinetic trapping mechanism — A family of synthetic riboswitches adopts a kinetic trapping mechanism — SUPPLEMENTARY DATA 

# A family of synthetic riboswitches adopts a kinetic trapping mechanism

## SUPPLEMENTARY DATA

**Files in this Data Supplement:**

- SUPPLEMENTARY DATA
