# Supplementary material for: A family of synthetic riboswitches adopts a kinetic trapping mechanism
Source: Nucleic Acids Res. 2014 Apr 29;42(10):6753–61. doi: 10.1093/nar/gku262 (PMC4041436; doi:10.1093/nar/gku262)
Supplement: SUPPLEMENTARY DATA [file supp_gku262_nar-03052-f-2013-File007.docx]

Supporting Information for

A Family of Synthetic Riboswitches Adopts a Kinetic Trapping Mechanism

Dennis M. Mishler and Justin P. Gallivan*

Department of Chemistry, Emory University, 1515 Dickey Drive, Atlanta, GA 30322

*To whom correspondence should be addressed.

email: justin.gallivan@emory.edu, phone: (+1) 404-712-2171, fax: (+1) 404-727-6586

**
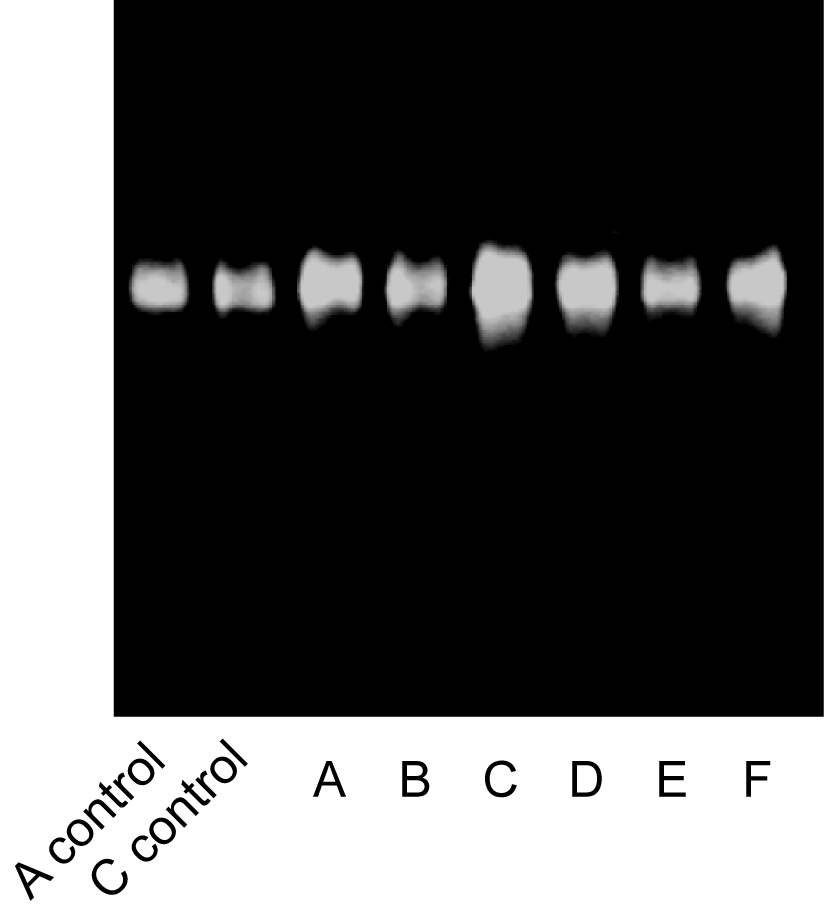
**

Figure S1) Agarose gel of RNA templates used in Figure 2B.

Luciferase mRNAs with riboswitch sequences were run on a 1% non-denaturing agarose gel using TBE. RNA was visualized using ethidium bromide and UV trans-illumination.


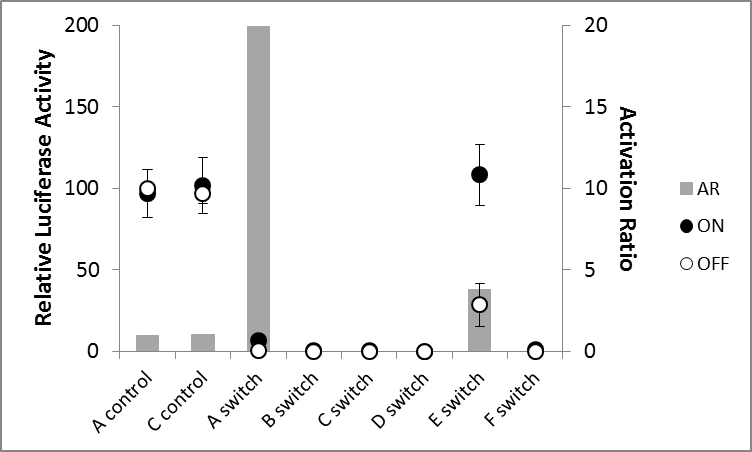


Figure S2) RNA templates heated in the presence of ligand and translated in extract.

The templates used in Fig 2B were heated to 65°C for 5 minutes in the presence or absence of 2 mM theophylline and then cooled at room temperature for 5 minutes prior to translation in S30 *E. coli* extract. Data are presented as in Figure 2. It is important to note that both luciferase controls as well as the E switch function as seen in Figure 2. Although heating in the presence of ligand can yield different results, there are two important factors to remember:

1. Most of these sequences have OFF state structures with very long stems (15-20 base pairs each, Figure S5);
2. When initially folding co-transcriptionally the theophylline aptamer is completely transcribed prior to the sequester sequence. However, in the above experiment the sequestering sequence is also present.

Additionally, the following facts support the interpretation that the RNA are not misfolded:

1. The only difference between A control and A switch is the theophylline aptamer.
2. The same is true for C control and C switch.
3. The only difference between E switch and the other riboswitch sequences is the sequence immediately adjacent to the theophylline aptamer (Figure S5).

Since our interests lie in understanding riboswitch function in biologically-relevant systems, rather than further pursuing how pre-transcribed RNA templates function in extract, we decided to focus on DNA templates that are transcribed and translated in extract. However, the results in Figures 2B and S2 are consistent with a model where most of our riboswitches attain a stable structure co-transcriptionally, either an ON or OFF state, and they are then trapped in this conformation.


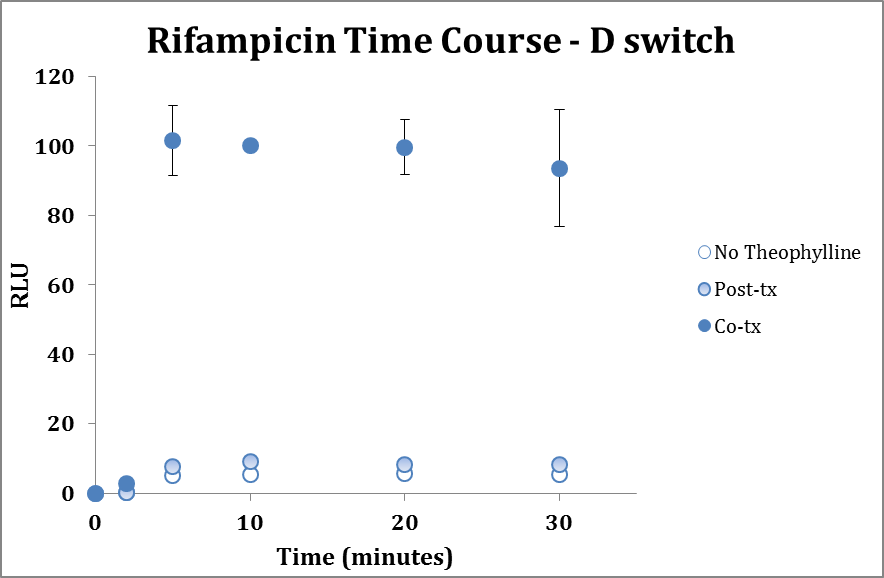

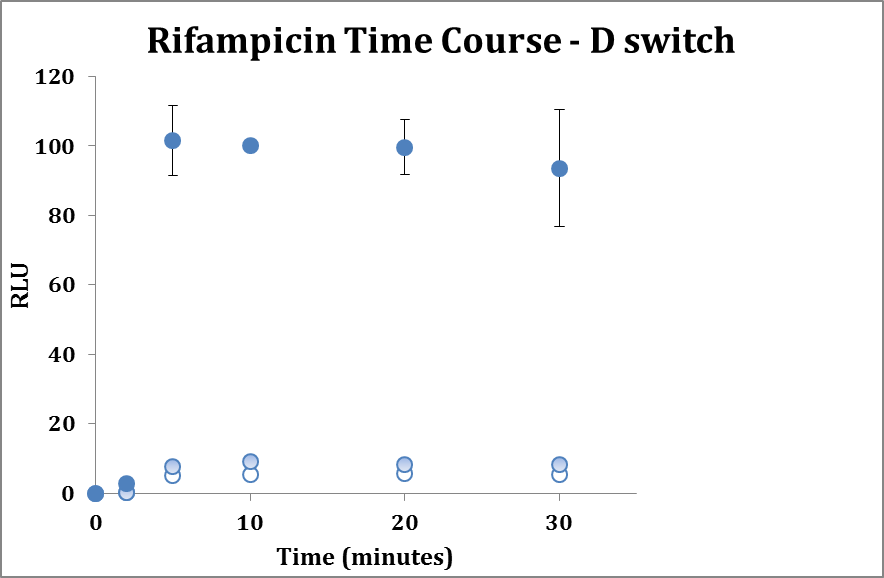


Figure S3) Decoupling transcription and translation in *E. coli* S30 extract.

Decoupled time courses for riboswitch D in S30 extract. The procedure and data are presented as in Figure 3D except that the translation period went for 30 minutes instead of 5 minutes. Data are normalized to the 10 minute co-transcriptional time point for consistency.

No theophylline added (open white circles), theophylline added only after transcription was terminated (faint blue circles), 2 mM theophylline present during transcription and translation (solid blue circles).

It is important to note that after 10 minutes there is little additional signal. Given that no new RNA is produced after addition of rifampicin (prior to time = 0), this is not surprising as the translation reaction is proceeding in *E. coli* extract

Figure S4) See caption on page S7.

Figure S4 continued) See caption on page S7.

Figure S4) Decoupled transcription and translation in extract.

Decoupled time courses for riboswitches A, B, C, E, and F in S30 extract. The procedure and data are presented as in Figure 3D: No theophylline added (open white circles), theophylline added only after transcription was terminated (faint blue circles), 2 mM theophylline present during transcription and translation (solid blue circles). Additionally, for the C riboswitch, a fourth condition: 500 uM theophylline present during transcription and 2 mM theophylline present during translation (dark blue circles) was used.

The C riboswitch is presented last as it does not conform to the same pattern that the other five riboswitches do. That pattern being that the increase of luciferase activity by post-transcriptional addition of theophylline is inversely correlated to the riboswitch’s observed activation ratio. The observed difference between the C riboswitch and the others is likely attributable to the architectural differences seen in the C riboswitch relative to the other riboswitches (Figure S3). It is possible that the C riboswitch’s different 5ʹ UTR structure could result in a transcriptional pause site, a different RNA folding landscape, or differences in RNA stability. Any of these options could explain why the post-transcriptional addition of theophylline could result in greater gene expression than we had otherwise expected.

A switch

B switch

D switch

C switch

Figure S5) See caption on page S9.


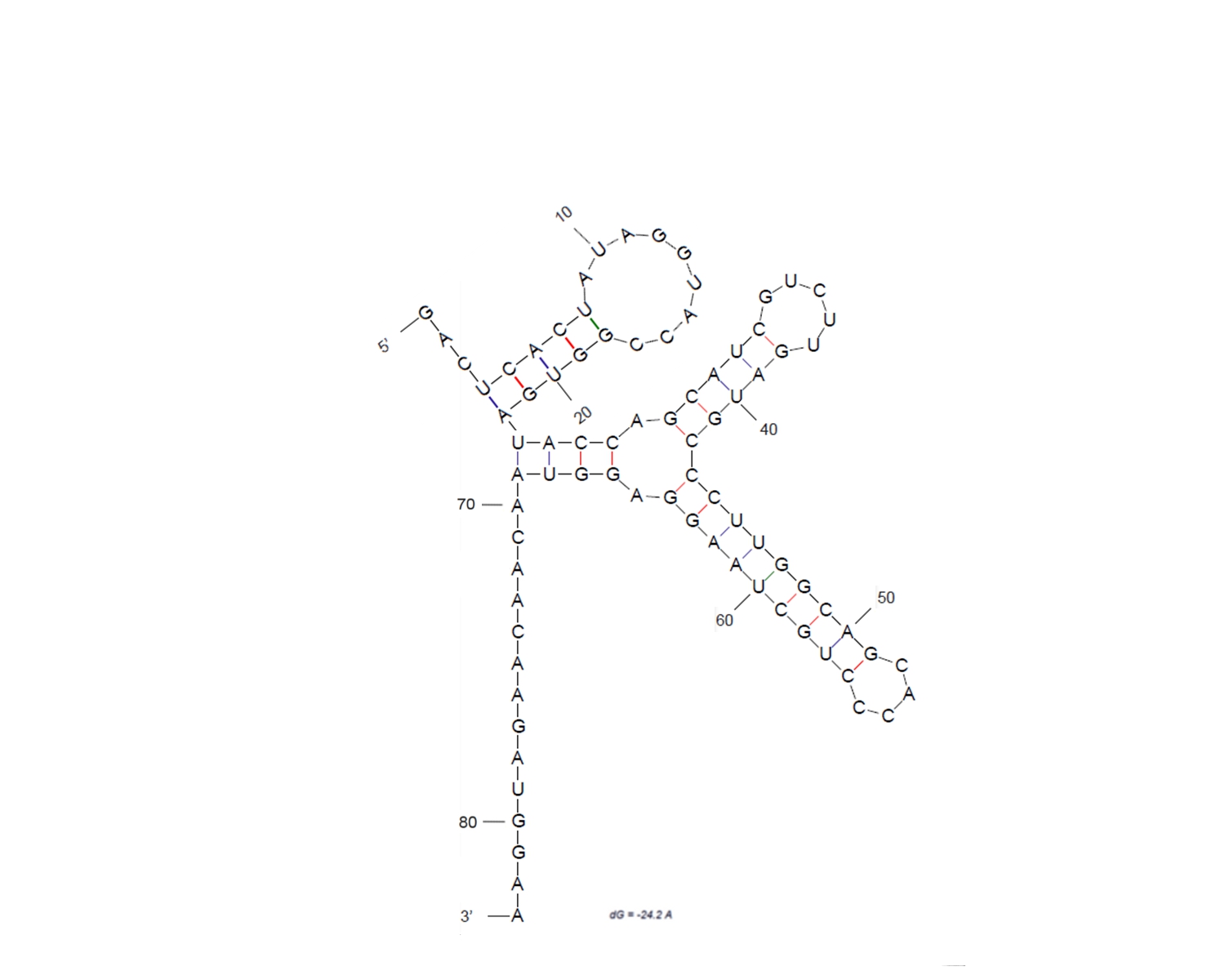


E switch

F switch

Figure S5) OFF state secondary structures and 5ʹ UTR sequences of Riboswitches A–F.

The OFF state structures were generated using mfold. The ΔG’s of the OFF states are presented below each structure.

Of important note are: the significantly more stable 5ʹ-terminal stem-loop of the C switch; and the relatively unpaired OFF state of the E switch, which explains the high levels of expression in the absence of theophylline.

Figure S6) OFF state secondary structures and 5ʹ UTR sequences for the D family riboswitches (shown below on pages S11 and S12) used in Tables 1 and S1 (page S14).

The OFF state structures were generated using mfold. The ΔG’s presented in Table 1 of the OFF states are presented below each structure. The eight variable nucleotides found in Table 1 are depicted here in lower case. The sequester helix is the stem loop structure that includes a portion of the aptamer and the RBS. This value is also shown in Table 1 and used in Figure 4.

615

611

612B

602A

607

D switch

602B

609

Figure S7) Luciferase assays in *E. coli* S30 extract using RNA templates.

RNA templates of the D family of riboswitches in Figure 4 and Table 1 with similar *in vivo* ON expression levels were added to extract. The procedure and data presentation are as in Figure 2B. Data are normalized to 602A with 2 mM theophylline. Each data point is the average of three independent experiments. Error bars show standard deviation.

Gene expression for the sequences with the most stable OFF states is greatly reduced relative to the riboswitches with less stable OFF states when in the presence of theophylline. Additionally, those riboswitches that had in vivo activation ratios ranging from greater to 40 to over 200 have activation ratios that are less than 10 when RNA templates are used.

| **Construct** | **OFF expression** | **ON expression** | **AR_1_** | **AR_2_** | **AR_3_** | **Avg AR** | **std** |
| --- | --- | --- | --- | --- | --- | --- | --- |
| 615 | 17 | 91 | 6 | 5 | 5 | **5** | 0.5 |
| 611 | 11 | 75 | 6 | 6 | 7 | **7** | 0.8 |
| 612B | 22 | 177 | 8 | 6 | 10 | **8** | 1.7 |
| 602A | 6 | 58 | 11 | 8 | 13 | **10** | 2.6 |
| D switch | 1 | 100 | 98 | 76 | 97 | **90** | 12.1 |
| 607 | 2 | 84 | 66 | 23 | 33 | **41** | 22.6 |
| 602B | 0 | 65 | 254 | 199 | 278 | **244** | 40.4 |
| 609 | 0 | 35 | 143 | 75 | 205 | **141** | 64.9 |

Table S1) *In vivo* luciferase data for Table 1 and Figure 4.

OFF expression is in the absence of theophylline. ON expression is in the presence of 2 mM theophylline. All data points were normalized relative to the D switch ON expression, which was set to 100. The purpose of displaying these values is to demonstrate that the ON expression levels are relatively similar for these constructs, being within 3 fold of D switch expression levels. These constructs are used in Figure S7.

The table also includes the estimated i*n vivo* activation ratios (ARs) for three independent cultures, performed on different days. These three ARs are then averaged together, giving the values used in Table 1 and Figure 4. Standard deviation for the three values is shown to the right, demonstrating some of the daily variability of estimating ARs, particularly for riboswitches with very low OFF expression levels.
